# Seismic noise in crystal neutron interferometry

**DOI:** 10.1107/S1600576725008660

**Published:** 2025-11-11

**Authors:** G. Mana, E. Massa

**Affiliations:** a INRIM – Istituto Nazionale di Ricerca Metrologica Strada delle Cacce 91 10135Torino Italy; Oak Ridge National Laboratory, USA; North Carolina State University, USA

**Keywords:** neutron interferometry, interference visibility, seismic noise, phase noise, split-crystal interferometry, interference fringes

## Abstract

We are involved in designing, constructing and operating a split-crystal interferometer that uses X-rays and neutrons simultaneously. Neutron interferometers are sensitive to seismic and acoustic noise due to the low speed, low flux and long detection time of thermal neutrons. The crystal splitting and the increased length and separation of the interferometer arms further heighten this sensitivity. To support the interferometer design and operation, we present an estimate of the root-mean-square phase noise when the interferometer is passively isolated from ground accelerations.

## Abstract 2

A split-crystal neutron interferometer allows longer arm separation and length. However, since the visibility of the interference is sensitive to seismic and acoustic noise, it is crucial to design and operate the interferometer acknowledging this problem. To aid in this process, we present a mathematical model that quantifies how time-dependent accelerations affect the phase and visibility of the interference fringes.

## 1. Introduction

Neutron and X-ray interferometry have been achieved with monolithic single-crystal interferometers first developed for X-rays by Bonse & Hart (1965 ▸) and later for thermal neutrons by Rauch and collaborators (Rauch *et al.*, 1974 ▸). Similarly to Mach–Zehnder interferometers in optics, a monochromatic X-ray or neutron beam is split through Laue diffraction, recombined using two mirror-like crystals and coherently mixed by the final crystal. Recently, observing neutron interference using separate crystals has led to the ongoing construction and operation of a skew-symmetric interferometer with extended arm separation and length (Lemmel *et al.*, 2022 ▸).

Matter-wave interferometers are inertial sensors whose sensitivity exceeds that of conventional mechanical and optical ones by many orders of magnitude (Clauser, 1988 ▸). Since the inception of neutron interferometry, it has been understood that the interference phase is sensitive to seismic and acoustic noise (Bauspiess *et al.*, 1976 ▸, 1978 ▸), and to accelerations (Bonse & Wroblewski, 1983 ▸, 1984 ▸). These sensitivities stem from the slowness of thermal neutrons and the extended travel time through the interferometer. New investigations and an interferometer geometry with reduced sensitivity to low-frequency noise are discussed by Pushin *et al.* (2009 ▸, 2011 ▸) and Nsofini *et al.* (2017 ▸).

We are engaged in a research project focused on designing, building and operating a split-crystal interferometer. In light of the cited observations and analytical findings, we have expanded the model of interferometer operation to account for time-dependent accelerations of the split crystals. The goal is to investigate how seismic and acoustic noise affect the phase and visibility of the interference fringes. Our work builds upon findings regarding gravity in crystal neutron interferometry (Sasso *et al.*, 2024 ▸; Massa *et al.*, 2024 ▸), which similarly apply to interferometry under the influence of accelerations. To take the time dependence into account, we utilize a novel approach first described by Sasso *et al.* (2024 ▸) that explains the effects of gravity and accelerations in terms of the crystals’ tilts and displacements observed from the neutron’s perspective.

The paper is structured as follows. Section 2 outlines the operation of the interferometer and the propagation of neutrons in the accelerated interferometer crystals. After discussing the visibility of the interference fringes in Section 3, we illustrate in Sections 4.1 and 4.2 how the phase noise and visibility are influenced by the power spectral density of the interferometer’s acceleration in both monolithic and split interferometers. The dependence of the interference-fringe visibility on the frequency of the seismic noise is examined in more detail in Appendix *A*[App appa].

All symbolic computations were made with the assistance of *Mathematica* (Wolfram Research, 2023*a* ▸); the relevant notebook is provided as supporting information. To view and interact with it, download the *Wolfram Player* free of charge (Wolfram Research, 2023*b* ▸).

## 2. Neutron interferometry

A description of the interferometer’s operation can be found in our previous paper (Sasso *et al.*, 2024 ▸), which extends Laue dynamical diffraction and the working of crystal interferometers to include the effect of gravity from first principles. The formalism, convention choices, symbols and notations employed there are strictly adhered to here. Below is a summary of the mathematics of interference. Here, we neglect gravity and the Coriolis force but assume the equivalence principle. This implies that the propagation of neutrons in a crystal under constant acceleration 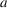 is equivalent to the neutrons being subjected to the gravitational force 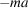, where *m* is the neutron mass.

**Figure 1:**
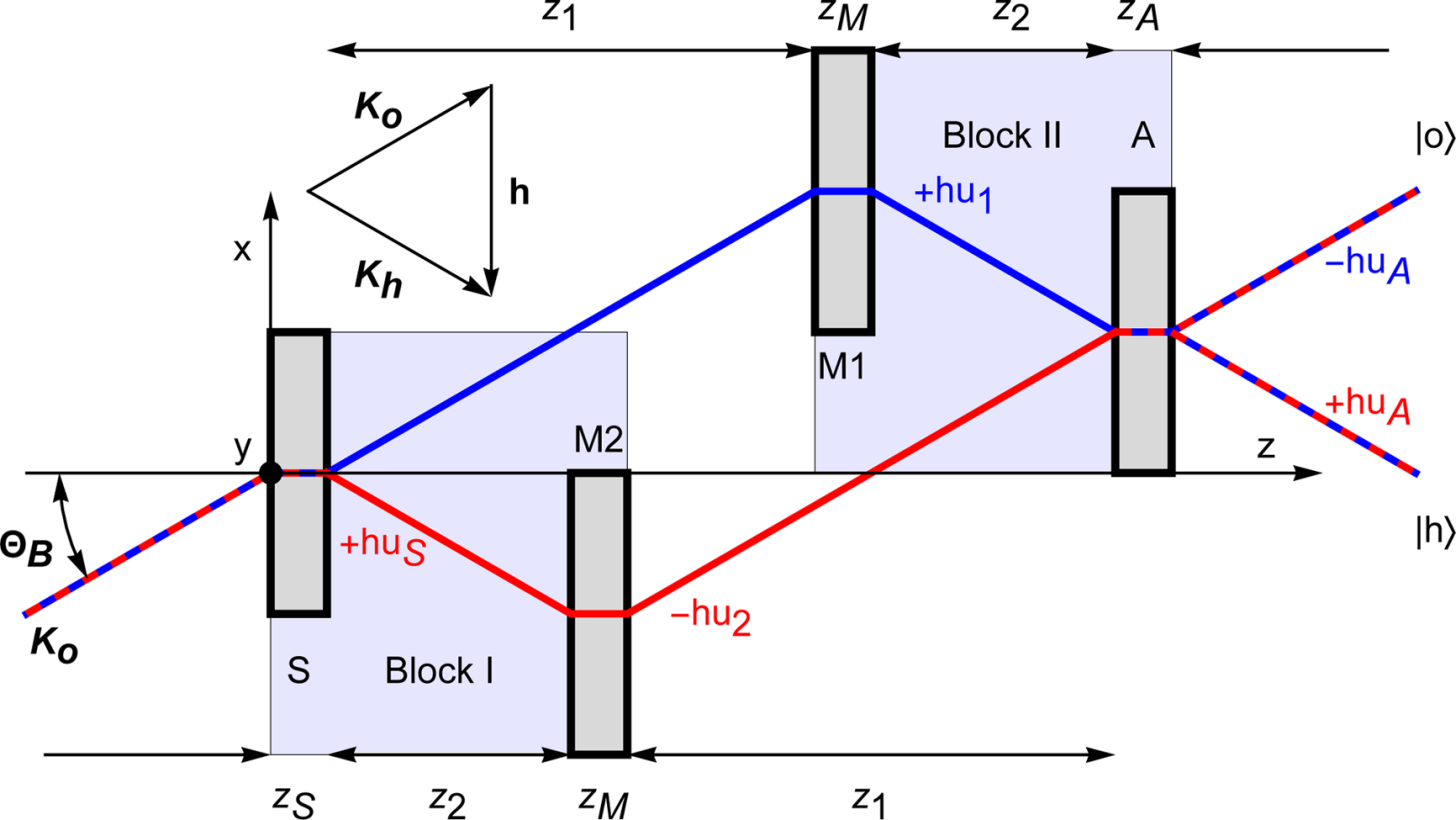
Top view of a skew-symmetric triple-Laue interferometer with separate crystals, blocks I and II. S = splitter, M1 and M2 = mirrors, A = analyser. The blue and red base rays indicate the first and second arms, respectively. The *x* and *y* axes are orthogonal to the diffracting and reflection planes, respectively. The *z* axis is orthogonal to the splitter, mirrors’ and analyser surfaces. The 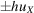 labels are the phases attained by the reflected state relative to the forward transmitted one.

Fig. 1 ▸ illustrates a split-crystal triple-Laue interferometer with a skew-symmetric and coplanar geometry. It also provides the meanings of some symbols that we will use. The interferometer comprises four symmetrically cut Si crystals (*i.e.* the diffracting planes are perpendicular to the surfaces), a splitter, two mirrors and an analyser. The first crystal splits a neutron beam into two beams, which are reunited in the final crystal with the assistance of two mirror-like crystals.

The *x* axis is opposite to the reciprocal vector **h**: 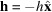. The optical *z* axis is perpendicular to the surfaces of the crystals. Together with the *y* axis, perpendicular to the *xz* reflection plane containing the 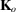 and 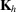 wavevectors, they form a right-handed frame. The *z* coordinate is a fictitious time, and it is related to time *t* by 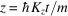, where 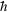 is the reduced Planck constant and 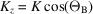 is the *z* component of 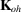. According to this interpretation, since we restrict our analysis to the reflection plane *xz*, neutrons move in one dimension along the *x* axis.

Each crystal produces two beams (transmitted and reflected) via Laue diffraction, which move forward in the 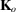 and 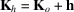 directions. Diffraction arises when the chief wavevectors of the beams, 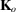 and 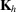, meet the Bragg conditions, that is when 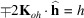 and 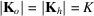.

We assume an ideal geometry that ensures complete visibility of interference fringes (see Fig. 1 ▸). Therefore, the splitter and analyser have the same thickness, denoted by 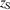 and 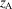, respectively (for a list of the main symbols used in this paper see Appendix *B*[App appb]). Hence, 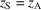. The same applies for the two mirrors, whose thicknesses are denoted by 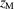. Next, the distance of mirror M2 from the splitter, 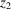, is equal to the analyser’s distance from mirror M1. Finally, the distance of mirror M1 from the splitter, 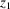, is equal to the analyser’s distance from mirror M2.

Regarding crystal thickness, two possibilities are worth considering: 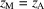 and 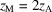. Here, we focus on the case 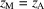. Furthermore, we assume that the split crystals are kept perfectly aligned by a feedback loop. Therefore, relative rotations are minimal, and this study will concentrate exclusively on crystal motions that are perpendicular to the diffracting planes, as well as on rotations about the vertical axis.

Each neutron within the interferometer can be represented by a quantum two-state system. Its superposed 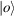 and 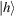 basis states propagate (in the fictitious time *z*) along the *x* axis in opposite directions, which are linked to the *x* component of the 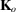 and 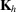 wavevectors.

Neutron interference is sensitive to linear accelerations orthogonal to the *yz* diffracting planes and angular acceleration about the normal to the *xz* reflection plane (Pushin *et al.*, 2009 ▸). Crystal propagation and interferometry in a uniformly accelerated frame are discussed by Bonse & Wroblewski (1984 ▸).

Pushin *et al.* (2009 ▸) approximate the Bragg reflection as an elastic bounce on a flat surface. This approximation holds if the Bragg condition is exactly satisfied. In general, since the momentum transfer is always perpendicular to the diffracting planes and (setting 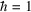) equal to 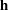, reflection is not specular (Lemmel, 2014 ▸).

A quantum-information approach to the dynamical diffraction theory has been applied to investigate the operation of a zero-area four-blade interferometer, which has been demonstrated to have a subspace that protects the interference visibility from low-frequency mechanical vibrations (Nsofini *et al.*, 2016 ▸, 2019 ▸; Nahman-Lévesque *et al.*, 2022 ▸).

We describe the operation of the interferometer under time-dependent accelerations from the perspective of the travelling neutrons (Sasso *et al.*, 2024 ▸). In the laboratory frame, the neutron motion is straight. They traverse the interferometer along the same paths they would follow if the interferometer was stationary, but propagate through accelerated crystals differently displaced. The transfer matrix that propagates neutrons through accelerated crystals is presented by Klink (1997 ▸) and is identical to that which propagates neutrons through a stationary crystal in a gravitational field (Sasso *et al.*, 2024 ▸).

To account for neutron propagation in a crystal accelerating along the normal to the diffracting planes (*x* axis), we omit the variations in acceleration during the crystal transit and assume that the crystal moves instead with a constant acceleration of 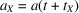, where *X* indicates the splitter (S), the mirrors (M1 and M2) or the analyser (A). Here, *t* is the arrival time at the splitter, 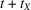 is the arrival time at the *X* crystal, and 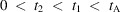 are the transit times from the splitter to the mirrors (M2 and M1) and analyser, respectively. No constraints apply to the separations of the crystals.

Therefore, neutrons propagate in motionless crystals and are assumed to be subject to the constant (inertial) force 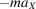. This approximation implies that the crystals are thin in relation to the acceleration dynamics. According to the Takagi–Taupin equations, propagation occurs in the same way as in a deformed crystal, where the diffracting planes are displaced along the *x* axis by half of the total crystal dis­placement during the transit, 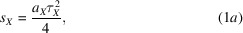
and tilted about the vertical by 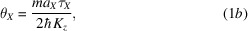
which is equal to half the propagation-direction change while guided by the crystal. Here, 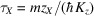 is the time of flight through the *X* crystal, *m* is the neutron mass, 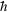 is the reduced Planck constant, 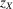 is the crystal thickness, 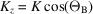 is the *z* component of 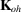, 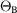 is the Bragg angle and we used 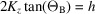. A detailed discussion of neutron propagation in accelerated crystals is presented by Sasso *et al.* (2024 ▸).

The Takagi–Taupin equations imply first-order approximations but the (inertial) acceleration affects the neutron motion quadratically. Therefore, the neutrons’ fall orthogonal to the diffracting planes is invisible. In particular, when the mean momentum meets the Bragg condition, crystals operate like a waveguide and neutrons propagate parallel to the diffracting planes (Sasso *et al.*, 2024 ▸). Consequently, the perceived motion stops and accelerated crystals drag neutrons with them. However, since it is a first-order effect, the inertial force 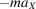 modifies the neutron velocity by 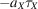, counteracting the change of the crystal velocity over the crystal-traversing time 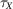. Consequently, the guiding by accelerated crystals does not affect the neutron velocity.

By using 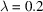
 nm neutron wavelength, silicon (220) diffracting planes and 2 mm thick crystals, the perceived tilt of the diffracting planes is 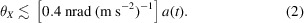
Therefore, provided the root-mean-square acceleration in the frequency band of interest — say from 1 mHz to 1 kHz — is less than 0.1 m s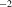, 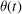 is negligible and the contribution to the neutron phase of the dynamical diffraction in the interferometer crystals will be neglected.

A displacement 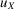 in a direction orthogonal to the diffracting planes of the *X* crystal alters the phase of the reflected neutrons (relative to the forward transmitted one) by 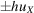. Assuming that plane waves are expressed as proportional to 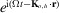, where 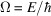 and *E* is the neutron energy, the phase advances with time *t* and retreats with (positive) propagation distance 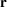. Therefore, the positive phase shift 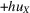 applies to neutrons incoming in the *o* state; the negative sign applies otherwise (see Fig. 1 ▸).

## 3. Phase noise

Assuming ideal geometry, the neutron flux at the 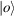 output port of the interferometer (see Fig. 1 ▸) is 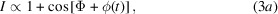
where Φ is the difference of the phases accumulated by the neutron along the two interferometer arms and 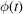 is a parasitic phase jitter, *e.g.* induced by seismic noise.

Due to the limited neutron flux and long integration time *T*, the phase jitter readily exceeds the detection bandwidth. Therefore, the detected flux is 
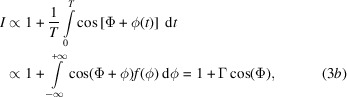
where 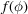 is the probability density function of the phase noise (assumed stationary and ergodic), and the interference visibility Γ will be compromised, *i.e.*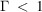.

**Figure 2:**
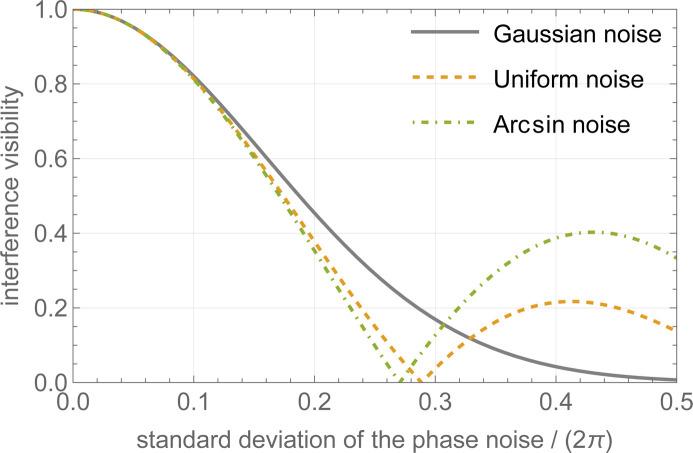
Visibility loss of the interference versus the standard deviation of Gaussian (solid line, grey), uniform (orange line, dashed) and arcsin (green line, dot–dashed) phase noise (bandwidth-limited and white).

For instance, assuming Gaussian noise with zero mean and 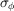 standard deviation, the detected interference signal is 
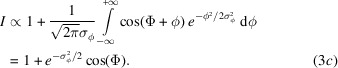
Fig. 2 ▸ shows the visibility loss due to an increasing phase jitter. Three probability density functions are compared: Gaussian, uniform and arcsin (see the supporting information).

To quantify the interference quality, the key metrics are the variance, 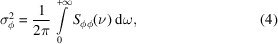
and (one-sided) power spectral density, 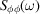 (which describes how the noise power is distributed over different harmonic components having angular frequency 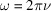), of the phase noise.

## 4. Seismic noise

The phase of single-particle interference is proportional to the sum, with signs, of the displacements of the interferometer crystals calculated at the neutron’s arrival at each of them. These displacements should be calculated backwards, starting from the arrival time at the detector. However, it is simpler to calculate them forwards, starting from the neutron’s arrival at the splitter. In so doing, the origin of the phase timescale is shifted by the time of flight from the splitter to the detector, which does not affect the sought power spectral density of the phase noise.

### 4.1. Monolithic interferometer

Let us begin by examining the simplest case of a monolithic interferometer, concentrating on linear accelerations along the *x* axis. Following this, we shall explore rotations about the vertical and consider a split-crystal interferometer.

**Figure 3:**
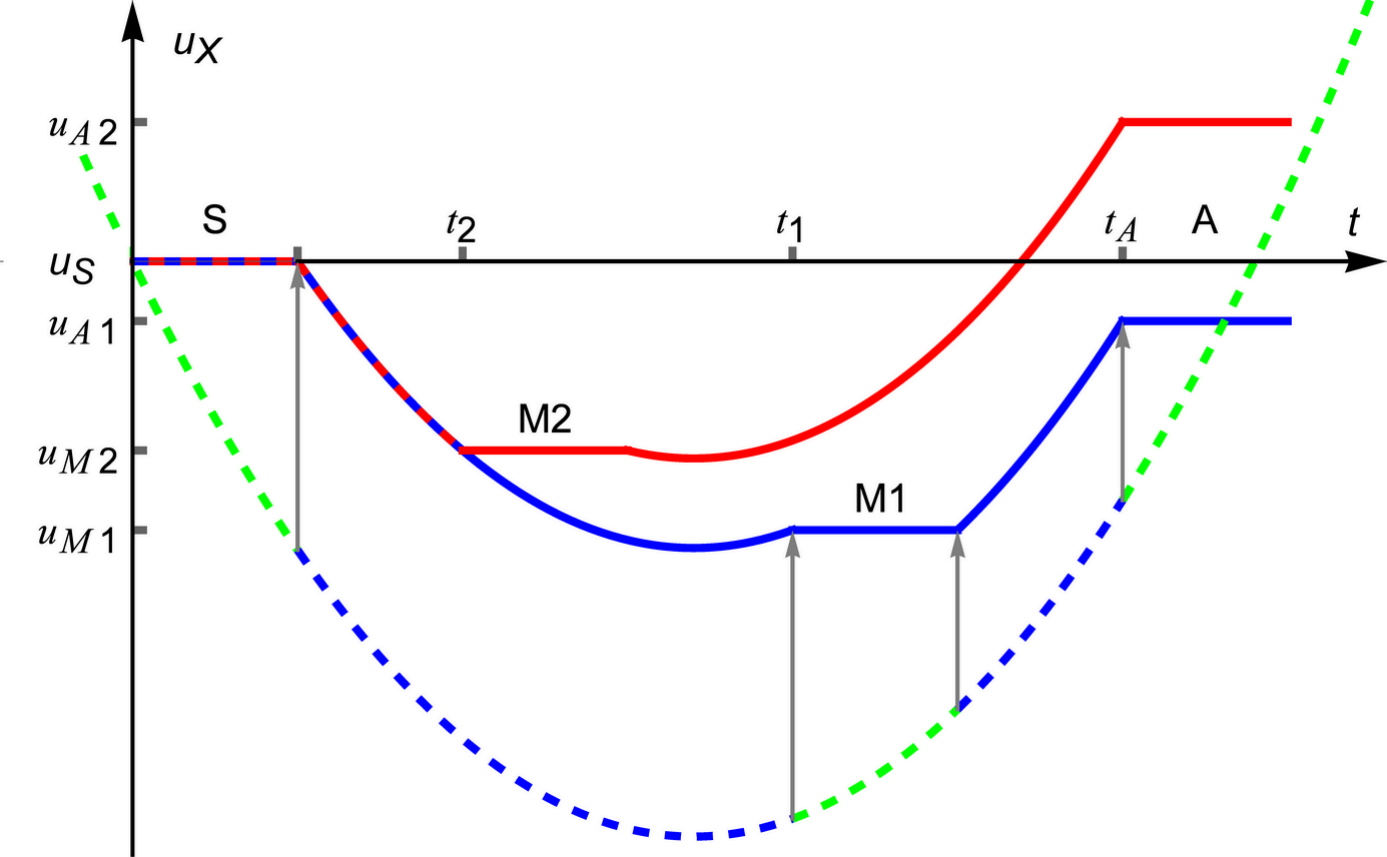
Monolithic interferometer. Displacements of the splitter (S), mirror (M1 and M2) and analyser (A) crystals as seen by a neutron entering the splitter at time 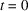 and traversing the interferometer along the first (blue line) and second (red line) arms. Since the moving crystals drag the neutron and the displacements related to the accelerated diffracting planes — see (1*a*) — have been omitted, the perceived displacements halt. The dashed (green) line is the interferometer motion. The arrows indicate the motion perceived by the neutron traversing the first arm. The neutron traversing the second arm has a similar perception.

From the neutron’s perspective, the accelerated crystals tilt and displace. Ignoring the tilts [see (1*b*) and (2)] and from the neutron viewpoint, three terms contribute to the displacement of the crystals, as shown in the subsequent equations (5*a*)–(5*c*) and Fig. 3 ▸. The first, 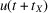, where *t* is the time when the neutron enters the splitter and 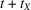 is the time it enters the *X* crystal, is the *X*-crystal displacement caused by the accelerated motion. The second term — in square brackets in (5*b*) and (5*c*) — is the neutron drag from the crystals previously traversed. Consequently, from the neutron’s viewpoint, it must be subtracted from the first term to obtain the *X*-crystal dis­place­ment perceived by the neutron. The last term is the diffracting-plane displacement 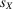 — see (1*a*) — related to the deformed crystal that is mathematically equivalent to the accelerated one.

The displacements of the splitter, mirrors and analyser seen by the neutron travelling along the 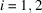 arms are indicated by 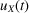 (where 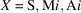 indicates the splitter, mirrors and analyser, respectively) and labelled by the same time, conventionally chosen to be that of the neutron’s arrival at the splitter. The phase noise 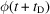 will be calculated at the time 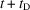, where 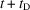 is the arrival time at the detector. Hence, 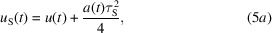

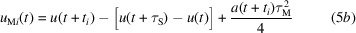
and 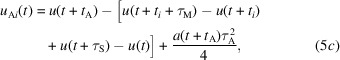
where 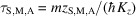 are the times of flight through the interferometer crystals, 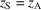 (splitter and analyser) and 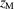 (mirrors M1 and M2) are the crystal thicknesses, and 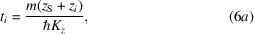

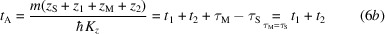
are the times of flight from the input surfaces of the splitter and *i*th mirror to that of the analyser (see Fig. 1 ▸).

Since the distance of mirror M2 from the splitter is equal to that of mirror M1 from the analyser, 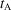 is independent of the interferometer arm travelled by the neutrons. However, due to the drag of the mirrors, as demonstrated by (5*c*) and Fig. 3 ▸, the perceived displacement of the analyser depends on the arm that has been traversed.

The difference between the phases accumulated along the interferometer arms is 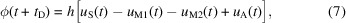
where, since the seen analyser displacement depends on the traversed arm, we heuristically averaged the two analyser displacements by setting 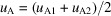.

**Figure 4:**
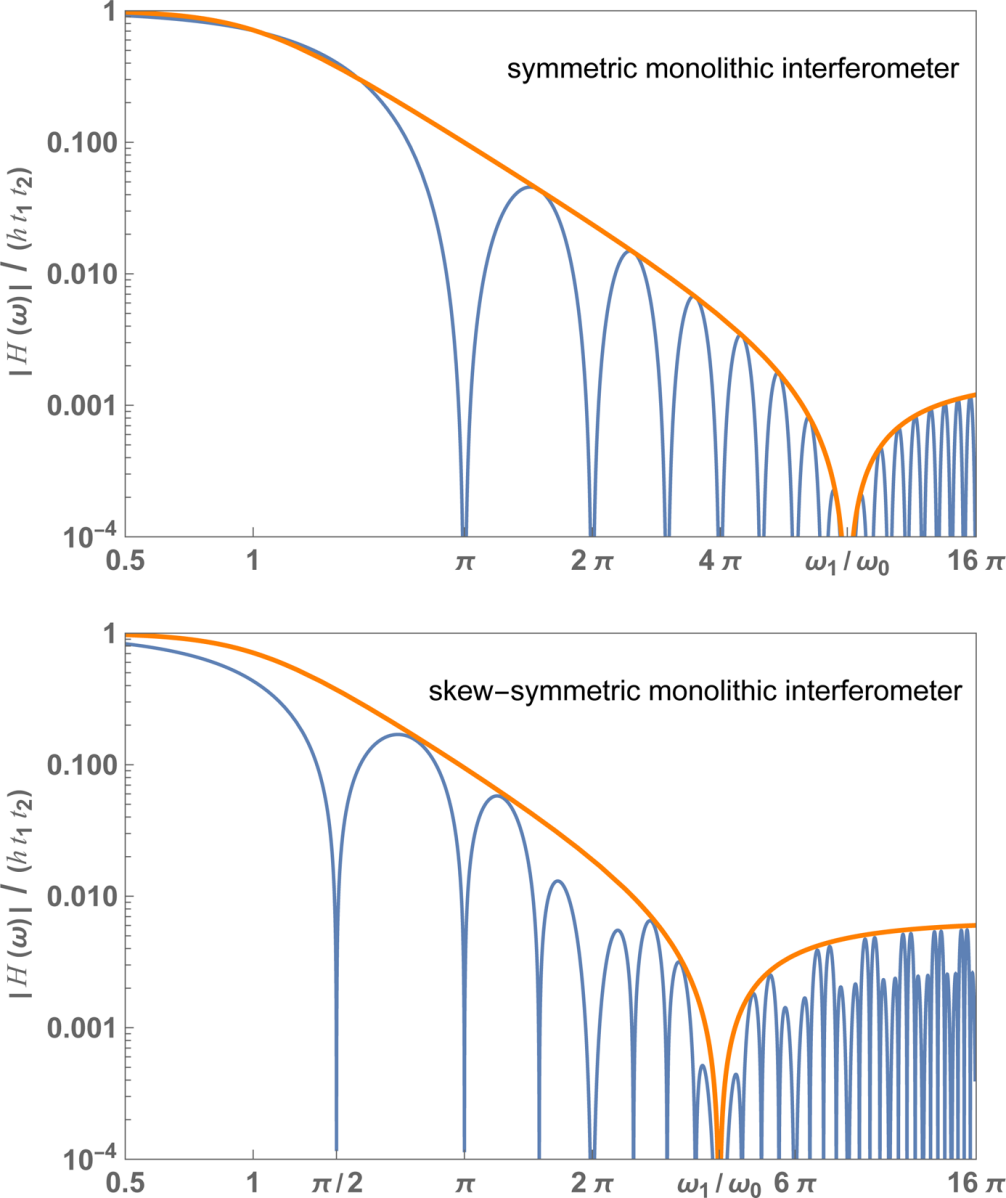
Monolithic interferometer. Blue: normalized magnitude of the frequency response of the interference phase to seismic noise versus the dimensionless (angular) noise frequency [see (11*b*)]. Orange: envelope [see (13*a*)]. Top: symmetric geometry, 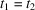. Bottom: skew-symmetric geometry, 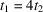. In both cases 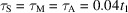. The crystal spacing and thickness were chosen to highlight the high-frequency behaviour of 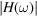. They are not representative of the interferometer design.

In the case of a uniform motion with constant velocity 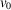, no phase difference is accumulated along the interferometer arms. In fact, if 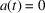 then (see the supporting information) 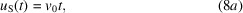

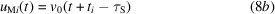
and 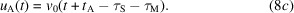
To investigate the effect of the seismic noise, we take the phase and acceleration Fourier spectra, 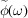 and 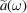, and calculate the Fourier domain representation of the transfer function, 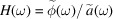. The phase spectrum is obtained by Fourier transforming (7) and observing that the spectra of the interferometer displacement, 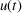, crystal displacements, 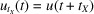, and interference phase, 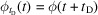, are 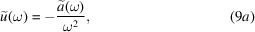

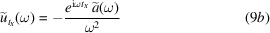
and 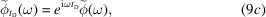
respectively (see the supporting information). Omitting the unessential phase factor and focusing on 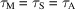, 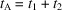 and thin crystals (that is, 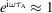 and 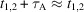), the transfer function, whose magnitude is shown in Fig. 4 ▸, can be approximated as (see the supporting information) 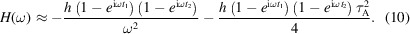
The first term encodes that, as neutrons traverse the interferometer, they see different crystal displacements, which are inversely proportional to the square of the acceleration frequency 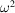 [see (9*b*)]. The second term encodes the different accelerations of the crystals, which, therefore, appear differently deformed, as shown in equations (1*b*) and (1*a*).

In the 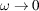 limit, the acceleration is constant and 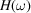 maps it in the Colella–Overhauser–Werner phase (Colella *et al.*, 1975 ▸). The 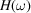 zeroes and maxima occur when the seen crystal displacements compensate or add constructively [see (7)]. This occurs at well defined frequencies, which depend on the neutron times of flight between the crystals.

The power spectral density of the phase noise is 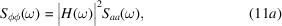
where 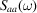 is the power spectral density of the seismic noise. The magnitude of the transfer function (see the supporting information) 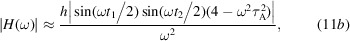
where we used 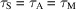 and the thin-crystal approximation, is shown in Fig. 4 ▸ for a symmetric (top) and skew-symmetric (bottom) interferometer.

The effect of a constant acceleration 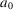 can be investigated by considering the limit of 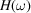 as ω tends to zero. Hence, 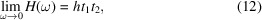
which, within the thin-crystal approximation made, encodes the phase induced by a static acceleration as that due to gravity in the Colella–Overhauser–Werner experiment (Sasso *et al.*, 2024 ▸).

Neglecting the crystal thickness, zeroes of 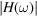 occur when 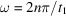 or 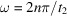 [see (11*b*)]. In fact, the times of flight between the interferometer crystals, 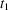 or 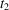, are an integer multiple of the oscillation period 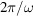. Therefore, 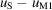 and 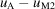, or 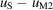 and 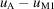, are null. This means that 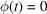 [see the supporting information and (7)].

In Fig. 4 ▸, the envelopes are the frequency responses of a linear system whose (similarly normalized) Laplace representation of the transfer function is (see the supporting information) 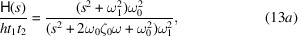
where 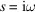 is the complex frequency, 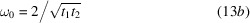
is the cutoff frequency of 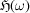 (indicated by 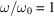 in Fig. 4 ▸) and the damping ratio 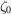 best fits 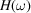. The zero value of 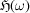, which occurs at 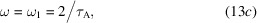
rules the transition from crystal-spacing to crystal-thickness dominations. The cutoff frequency makes a 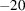
 dB per decade attenuation. The 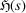 zero at 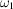 makes a 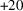
 dB per decade amplification and horizontally has the high-frequency asymptote 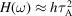.

### 4.2. Split-crystal interferometer

In a split-crystal interferometer (see Fig. 1 ▸), the two interferometer blocks rest on separate mechanical stages to allow for electronic control of their relative alignment and, as a result, may move independently. Consequently, we denote the motions of each block by 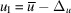 and 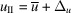, where 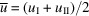 and 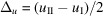 are the common (unison) and differential (counter-phase) motions, respectively.

Rotations about the vertical originate from counter-phase motions, which are included in 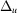. For the sake of simplicity, we neglect the difference of the rotationally induced dis­placements of the crystal pairs (splitter and mirror M2, mirror M1 and analyser) belonging to the same block.

**Figure 5:**
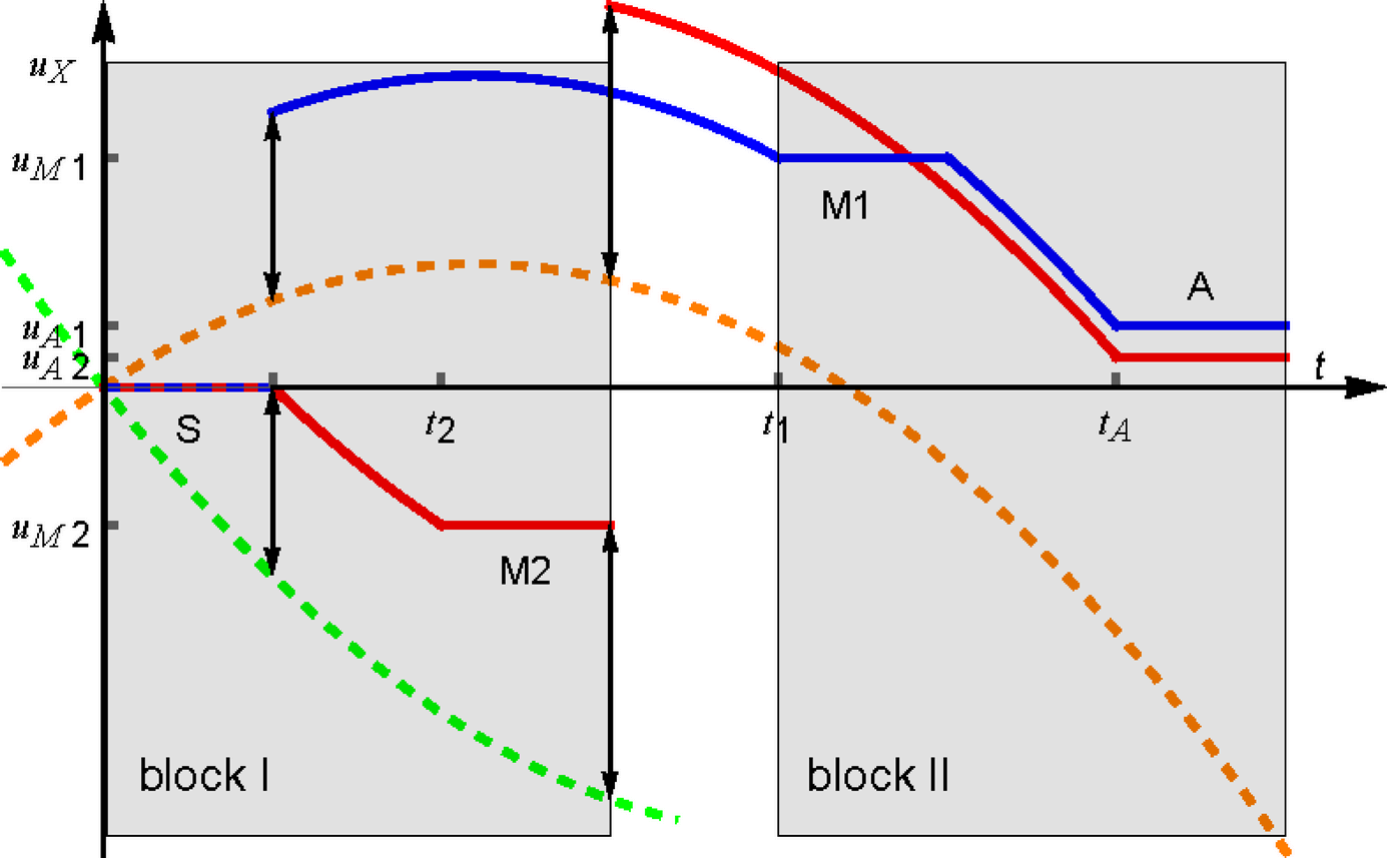
Split-crystal interferometer. Displacements of the splitter (S), mirrors (M1 and M2), and analyser (A) as seen by a neutron entering the splitter at time 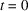 and traversing the interferometer along the first (blue line) and second (red line) arms. Since the moving crystals drag the neutron and the displacements related to the accelerated diffracting planes — see (1*a*) — have been omitted, the perceived displacements halt. The dashed lines are the motions of the first (green) and second (orange) block, which are made to coincide at 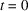. The arrows indicate the jumps of the second block perceived (because of the crystals’ drag) when the neutron leaves the first.

As done in Section 4.1, the displacements 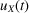 are labelled by the same time, conventionally chosen as that of the neutron’s entrance into the interferometer, and the phase noise will be calculated at the time 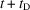. The perceived crystal displacements (see Fig. 5 ▸) are 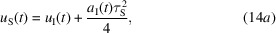

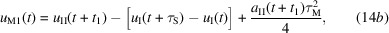

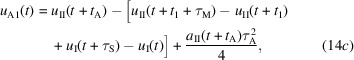

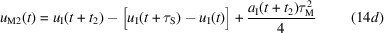
and 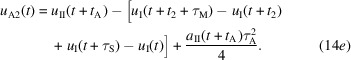
As before, the first term in (14*a*)–(14*e*) is the displacement 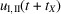 of the *X* crystal due to accelerated motion. The second term in (14*b*)–(14*e*) (in the square brackets) accounts for the drag exerted by the previously crossed crystals. As shown in Fig. 5 ▸, the drag has different effects depending on whether or not the previously traversed crystal is in the same block. In the first case, the perceived displacement comes to a standstill. In the second, it aligns with the differential motion of the two interferometer blocks. The final term of (14*a*)–(14*e*) is once more the perceived deformation of the accelerated diffracting planes [see (1*a*)]. Finally, the mutual displacement of the split crystals is irrelevant.

In the case of uniform motions with constant velocities 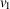 and 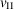, the block displacements are 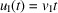 and 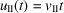. Since the displacement between the two blocks is irrelevant, the difference between the phases accumulated along the interferometer arms (see the supporting information), 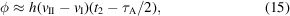
where we used 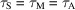, depends on the velocity difference and the separation between interferometer arms, which is proportional to 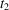. When 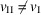, the phase difference originates because the neutrons see a difference between the relative displacement of the crystals in block I (splitter and mirror M2, see Fig. 1 ▸) and that of the crystals in block II (mirror M1 and analyser, see Fig. 1 ▸).

To investigate the case when 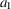 and 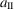 are constants, we set 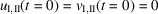 and assume 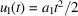 and 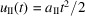. Neglecting the crystal thicknesses, from (14*a*)–(14*e*), we obtain (see the supporting information) 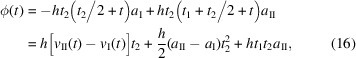
where 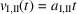. In (16), the terms proportional to *v*_II_(*t*) − *v*_I_(*t*) and *a*_II_ − *a*_I_ encode the difference between the relative displacements of the crystals in the first and second block (see Fig. 1 ▸). Because of the increasing difference, the phase difference depends on time and grows without limit. The last term, 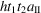, is consistent with the thin-crystal approximation (12) and the gravitationally induced phase in the Colella–Overhauser–Werner experiment (Colella *et al.*, 1975 ▸; Sasso *et al.*, 2024 ▸).

After Fourier transformation of (14*a*)–(14*e*) (see the supporting information), the Fourier spectrum of the phase noise (7) is given by 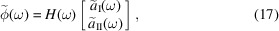
where 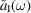 and 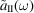 are the Fourier spectra of the first and second block accelerations, respectively. Using 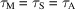, 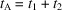 and thin crystals (that is, 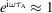 and 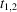
 
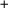

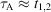
), 
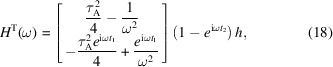
where the superscript T indicates the transpose. Similarly to the case of a monolithic interferometer, the terms proportional to 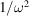 originate from the different crystal displacements. The terms independent of ω and proportional to 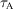 arise from the different (effective) crystal deformation.

The power spectral density of the phase noise is given by (see the supporting information) 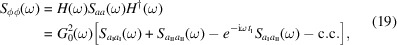
where the dagger indicates the conjugate transpose, c.c. is the complex conjugate of the preceding term,
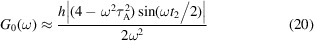
and 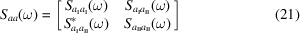
is the (multivariate) power spectral density of the seismic noise. The elements of *S_aa_*(ω) are the power spectral density and cross power spectral density of the accelerations.


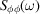
 depends on the split-crystals’ separation 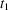 only through the correlated or anti-correlated motions of the split crystals, which is encoded in the cross power spectral density 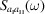. It can be rewritten as 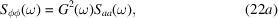
where 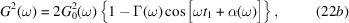

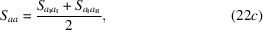

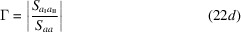
and 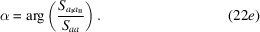
If the split crystals move in unison, then 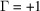 and 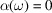. Since 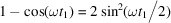, the frequency response (22*b*), 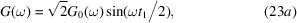
replicates the monolithic interferometer case [see (11*a*), (11*b*) and Fig. 4 ▸] and 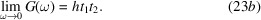
If the crystal motions are anti-correlated, then 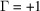 and 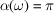 or, equivalently, 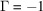 and 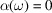. Therefore, in (22*b*), 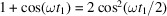, 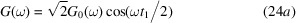
and 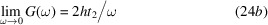
diverges, which seems counter-intuitive.

In fact, if 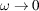, the crystals might seem not to move so much and, therefore, produce less phase difference. The origin of this divergence resides in the 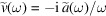 relationship between acceleration and velocity. Therefore, from (15), 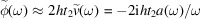. The 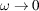 limit corresponds to constant and opposite accelerations, and 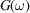 describes crystals having opposite and (steady-state) infinite velocities.

If the crystal motions are uncorrelated, then 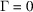 and 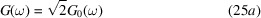
depends only on 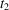, which is proportional to the separation of the interferometer arms. Also in this case, the low-frequency response of the interference phase to the acceleration, 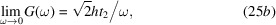
diverges. The divergence as the frequency tends to zero originates again from the linear increase of the split-crystals’ velocities; these velocities — since the accelerations of the split crystals are assumed to be uncorrelated — have a 50% probability of being opposite.

**Figure 6:**
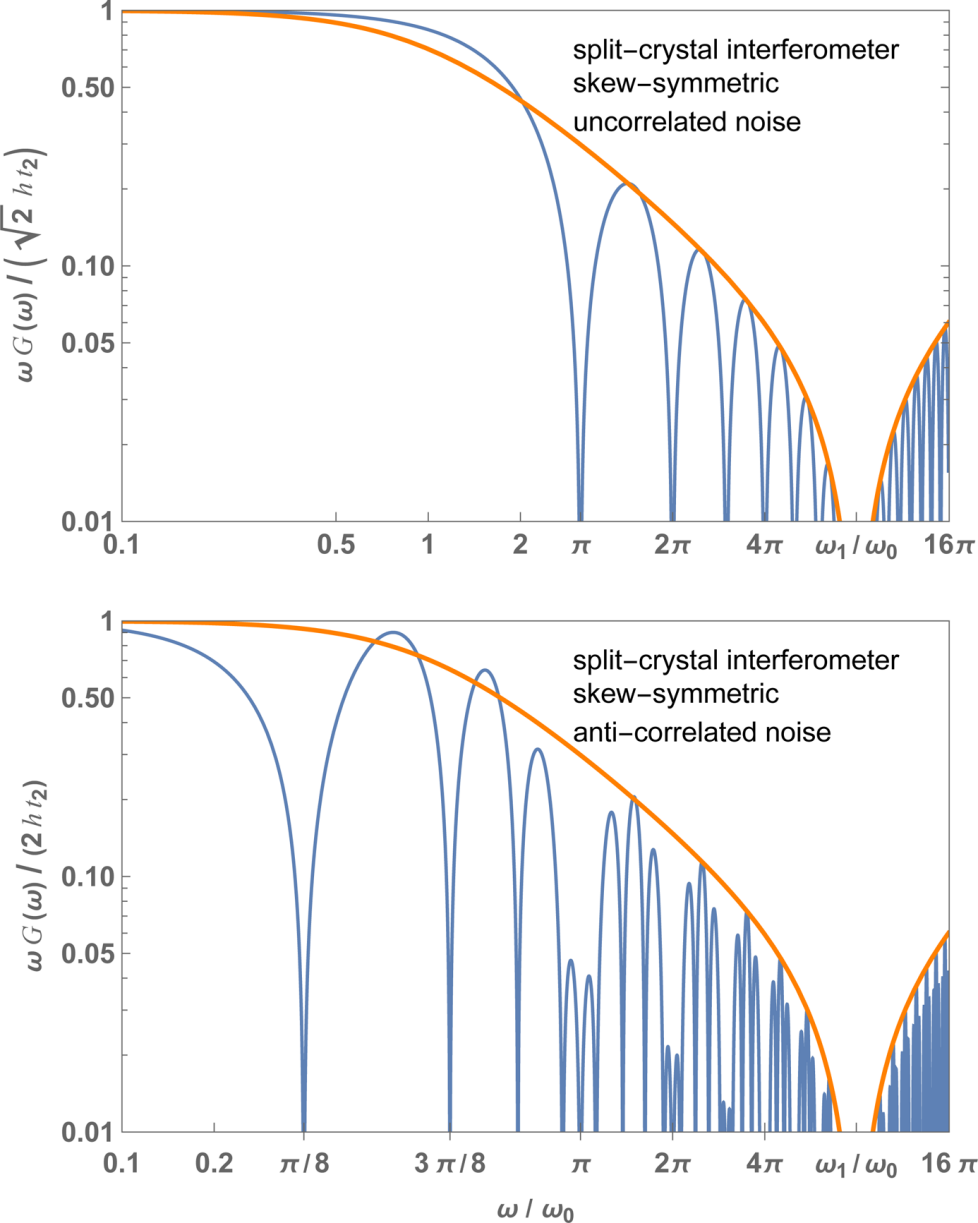
Skew-symmetric split-crystal interferometer. Blue line: normalized magnitude of the frequency response of the interference phase to uncorrelated (top) and anti-correlated (bottom) seismic noises versus the dimensionless (angular) noise frequency [see (20), (25*a*) and (24*a*)]. Orange lines: envelopes [see (26*a*)]. In both cases 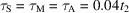, 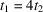 and 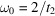. The crystal spacing and thickness were chosen to highlight the high-frequency behaviour of 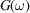. They are not representative of the interferometer design.

Fig. 6 ▸ shows the normalized frequency responses 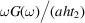, where 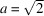 (uncorrelated noise) or 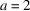 (anti-correlated noise), versus the dimensionless (angular) frequency of uncorrelated (top) and anti-correlated (bottom) seismic noise. The divergence in the 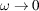 limit has been removed by the 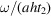 normalization factor.

Similarly to the monolithic interferometer case, the 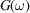 zeroes and maxima occur when the seen crystal displacements sum to zero or add constructively, respectively, which happens at well defined frequencies depending on the neutron times of flight between the interferometer crystals.

The envelopes are the frequency responses of a linear system whose (similarly normalized) Laplace representation of the transfer function is 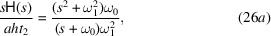
where *s* is the complex frequency, 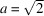 (uncorrelated noise) or 2 (anti-correlated noise), 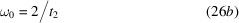
is the cutoff frequency and 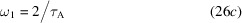
rules the transition from crystal-spacing to crystal-thickness dominations.

## 5. Numerics

We are involved in a research project that aims to operate a split-crystal interferometer at the S18 beamline of the Institut Laue–Langevin (ILL). The sensitivity of the interferometer to seismic and acoustic noise conflicts with the operations at ILL’s high-flux reactor, which necessitate a considerable amount of heavy machinery. Consequently, the seismic characteristics of the beamline resemble those of an industrial environment more than those of a metrology laboratory. The challenge is to establish an instrumental facility with a low level of vibration noise.

**Figure 7:**
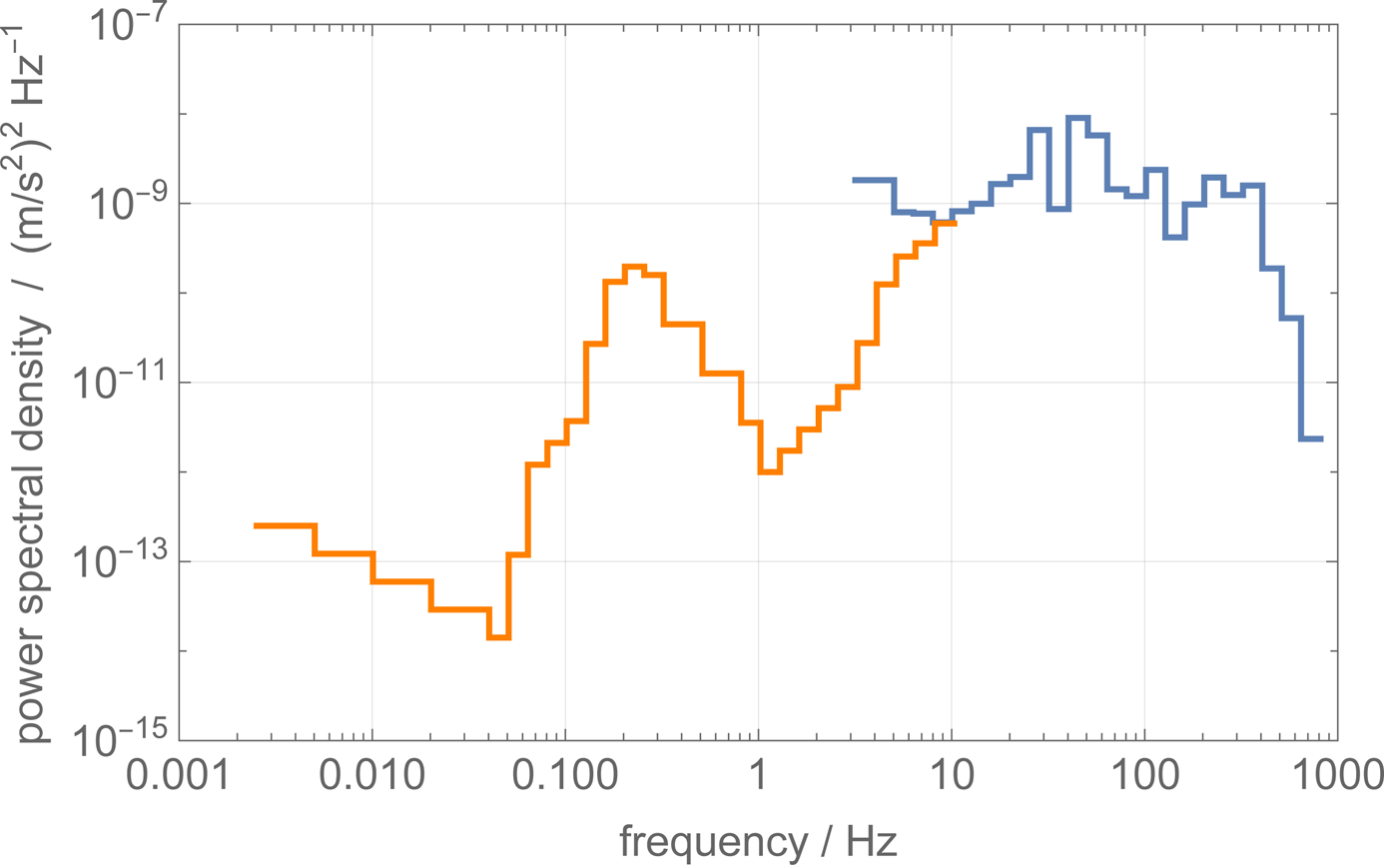
Blue: 1/3 octave power spectral density of the floor acceleration at ILL’s S18 beamline. Orange: Peterson’s high-noise model of the power spectral density of the vertical acceleration at the surface of the Earth.

Fig. 7 ▸ (blue line) illustrates the power spectral density of the floor (vertical) accelerations at the ILL’s S18 beamline. It was measured using a high-sensitivity accelerometer (Bruel and Kjaer type 8306). Investigating the low-frequency part of the spectrum was not possible. Hence, in the figure, we report the upper acceleration values using Peterson’s new high-noise model (orange line) (Peterson, 1993 ▸). The dominant feature is a peak in the frequency range from 0.1 to 1.0 Hz, generated by North Atlantic and Mediterranean Sea waves travelling in opposite directions and having equal periods, which produce gravity standing waves and a pressure perturbation that propagates to the ocean bottom (Marzorati & Bindi, 2006 ▸). The high-frequency part of the spectrum shows the manmade activities, which are the primary sources of noise, stemming from traffic and machinery. The cutoff near 1 kHz results from the accelerometer’s mounting and the low-pass filter incorporated in the built-in preamplifier.

According to Fig. 2 ▸, to prevent loss of visibility, the root-mean-square noise of the interference phase must be restricted to 10% of the fringe period for frequencies starting from 0.02 Hz (the typical duration of the neutron count is 50 s). The interferometer will be located inside a vacuum chamber to achieve sub-nanoradian resolution through optical interferometry, thereby ensuring control of crystal alignment. Consequently, acoustic noise will not present a problem.

**Figure 8:**
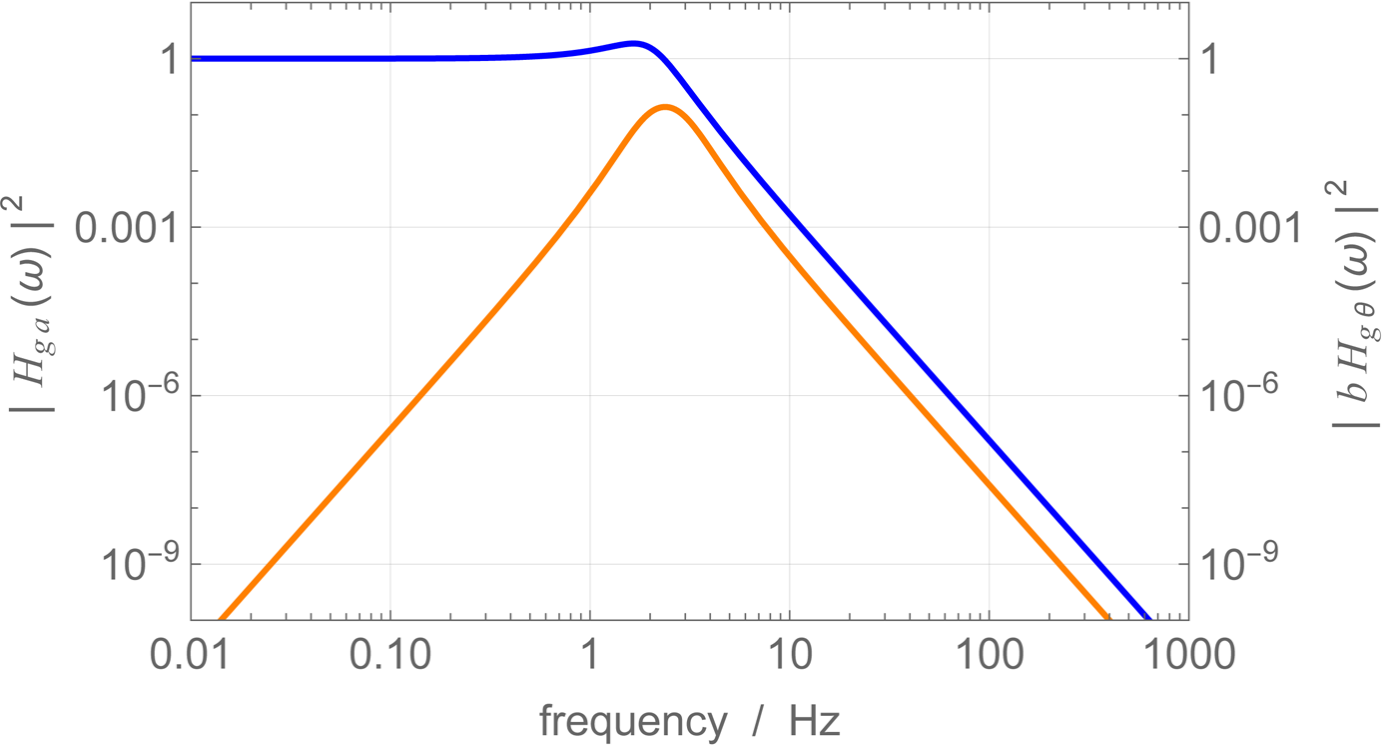
Mass–spring isolation system having 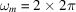 Hz linear resonance frequency and 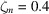 damping ratio. Blue: squared transmissivity of the ground acceleration. Orange: squared transmissivity of the ground acceleration to the mass angular acceleration. The angular resonance frequency is 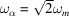. The radius of gyration is 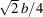, where *b* is the spring separation.

A passive vibration isolation system will be implemented to reduce ground accelerations. A typical installation comprises a mass (which may weigh up to a thousand kilograms) that rests on passive or active supports and acts as a damped oscillator with a resonance frequency of a few hertz. If 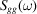 designates the power spectral density of the ground accelerations and 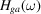 the transfer function from the ground to the interferometer, then the power spectral density of the interferometer accelerations is given by 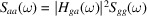. The transmissibility 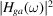 of an idealized isolation system (a mass–spring oscillator) is shown in Fig. 8 ▸ (blue line).

Since the asymmetries in the payload distribution and the responses of the isolators couple horizontal motions and twists, we must take angular accelerations about the vertical into account. If 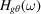 denotes the transfer function from the ground to the interferometer, the power spectral density of the angular accelerations is given by 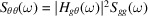. The 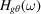 transfer function for a mass suspended by two springs is derived in the supporting information.


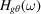
 maps linear accelerations to angular ones. Therefore, its unit of measurement is the inverse metre. The transfer function that converts ground acceleration into counter-phase accelerations of the split crystals is given by 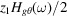, where 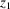 is the crystal distance. Assuming a 10% coupling and a gyration radius equal to 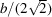, with *b* being the support separation, the transmissibility 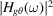 of the vibration isolation system is as illustrated in Fig. 8 ▸ (orange line). The disappearance of both high- and low-frequency noise components is attributed to inertia and the fact that steady-state linear acceleration cannot induce angular accelerations in the interferometer (see the supporting information).

Even fairly rigid supports and alignment devices for the split crystals exhibit their first resonances within the 100–500 Hz frequency range. Therefore, in the high-frequency domain, we anticipate uncorrelated motions of the split crystals, primarily due to differing frequency responses and resonances. In the low-frequency domain, we expect correlated and anti-correlated motions as a result of linear and twist accelerations of the vibration isolation table.

The design parameters of the split-crystal interferometer are presented in Table 1 ▸. Due to the extremely high cutoff frequencies 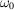 and 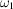, the interferometer transmissibility 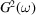 of ground acceleration to the interference-fringe phase can be effectively approximated by 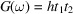 (in-phase motion), 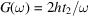 (counter-phase motion) and 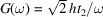 (uncorrelated motion).

By taking a constant power spectral density of the ground noise equal to 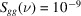 (m s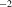)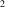 Hz^−1^, where 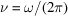 (see Fig. 7 ▸), the expected standard deviations of the phase noise are 
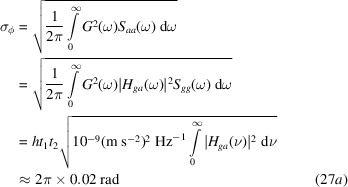
for the in-phase motions [see (22*b*) and (23*b*)], 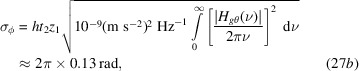
where the spacing between the table-top supports is assumed equal to 2 m, for the counter-phase motions [see (22*b*) and (24*b*)] and 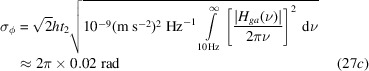
for the uncorrelated motions.

In the uncorrelated-motion case, as 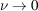, 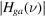 is constant while 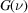 diverges [see (25*b*)]. However, at low frequencies, interferometer accelerations are expected due to the rigid motions (translations and rotations) of the entire setup. For this reason, the integration in (27*c*) starts from 10 Hz, which is well below the lowest resonance of the setup.

In-phase and uncorrelated oscillations are conveniently limited by standard isolation systems. In contrast, counter-phase oscillations associated with angular accelerations are critical because the transfer function mapping anti-correlated accelerations into phase noise diverges at low frequencies. To err on the side of caution, we conservatively assumed a constant power spectral density for ground accelerations at low frequencies, equal to the average power spectral density observed at higher frequencies, where ILL’s activity is a significant noise source.

## 6. Conclusions

A split-crystal neutron interferometer is being developed for operation at the S18 beamline of the Institut Laue–Langevin. Understanding the impact of gravitational force in terms of crystal displacements and tilts, as observed by free-falling neutrons in the work of Sasso *et al.* (2024 ▸), enabled us to consider time-varying and different (in-phase, counter-phase and uncorrelated) accelerations of the two blocks of the interferometer and investigate the effects of seismic noise on its operation. Once the power spectral densities of the crystals’ accelerations are available, the computation of the power spectral density of the interference-fringe phase is carried out using transfer functions that map the accelerations into the phase.

These transfer functions demonstrate cutoffs at angular frequencies that are inversely proportional to the time of flight of neutrons travelling from the first to the second block of the interferometer (in-phase accelerations) or to the time of flight between the crystals in the two blocks (uncorrelated and counter-phase accelerations). The cutoffs are followed by oscillations caused by in-phase and counter-phase movements of the crossed crystals, along with a constant high-frequency response resulting from propagation within the accelerating crystals. Unless the separation and length of the interferometer arms are substantial, the relevant transfer functions can be approximated as constants for all practical purposes.

Measurements of floor accelerations and the upper bounds predicted by Peterson’s new high-noise model have been used to estimate the root-mean-square phase noise when operating the interferometer on an optical bench that is passively isolated from ground seismic noise. This model illustrates the scenario expected when operating the interferometer. While in-phase and uncorrelated oscillations of the split crystals are effectively limited by isolation from seismic and acoustic noise present in the optics, counter-phase oscillations related to angular accelerations of the interferometer are critical and warrant attention.

## Supplementary Material

Mathematica 14.1 notebook. DOI: 10.1107/S1600576725008660/ei5136sup1.nb

## Figures and Tables

**Figure 9:**
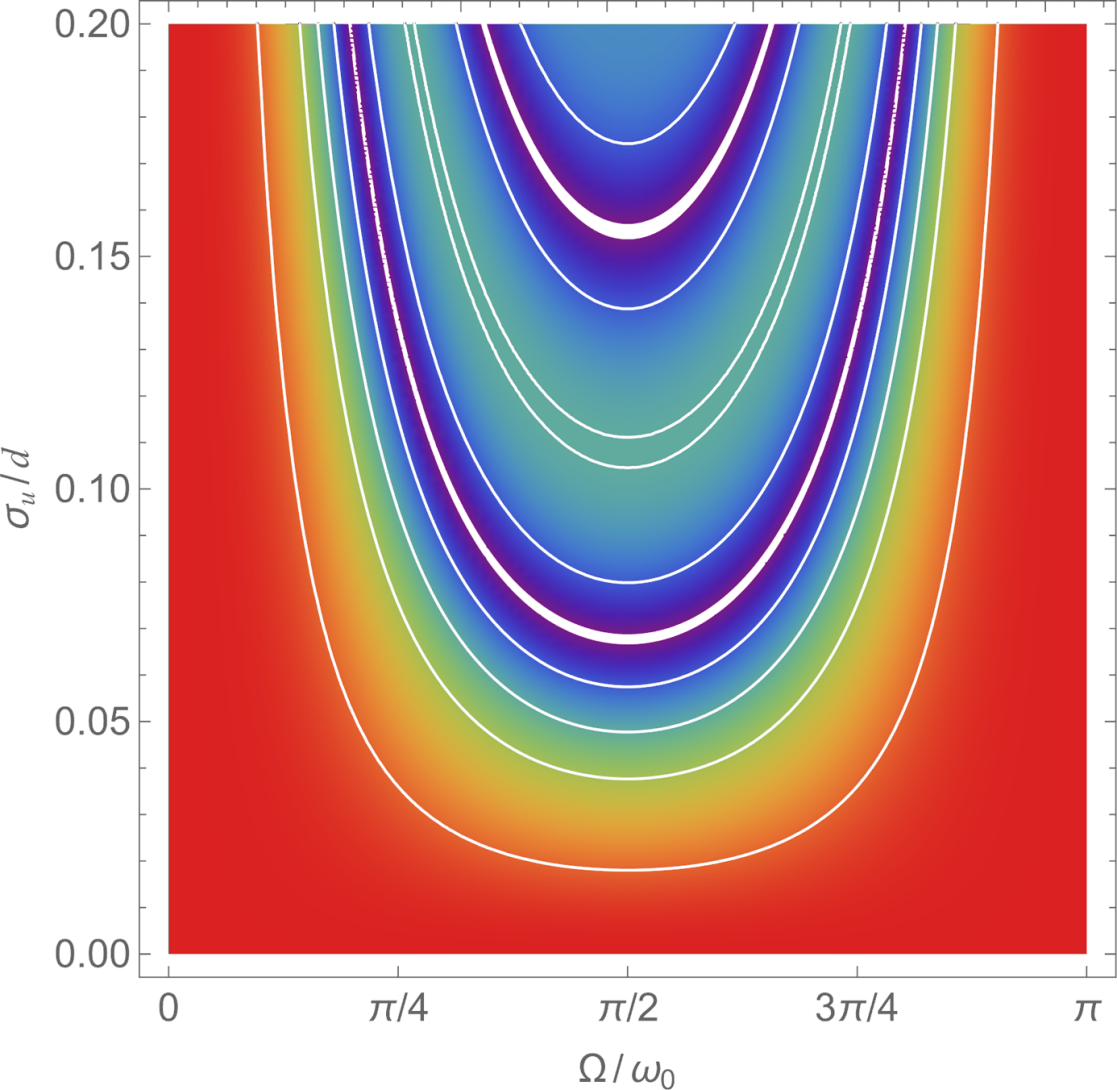
Monolithic and symmetric interferometer. Visibility Γ — see (35) — of the interference fringes versus the standard deviation 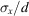 of a sinusoidal displacement having frequency 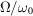. Colours run from one (red) to zero (violet). The contour lines indicate the values 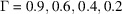 and 0.

**Table 1:** Design parameters of the split-crystal interferometer The diffracting planes are Si 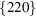. The two 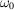 values are for correlated and uncorrelated or anti-correlated motions.

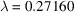 nm	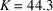 rad nm^−1^
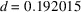 nm	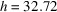 rad nm^−1^
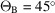	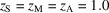 mm
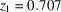 m	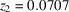 m
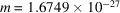 kg	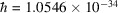 J s
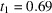 ms	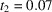 ms
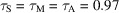 µs	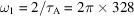 krad s^−1^
Symmetric geometry	Skew-symmetric geometry
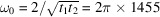 rad s^−1^	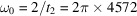 rad s^−1^

## Appendix A Fringe visibility

To examine the dependence of the interference visibility on the frequency of the seismic noise, let us assume noise having (two-sided) power spectral density

where  is the noise variance.

Referring to a monolithic and symmetric interferometer [see (11*a*), (11*b*) and Fig. 4 ▸ (top)], the power spectral density of the phase noise is

where we used  and omitted the  term because  for all practical purposes. *Mutatis mutandis*, the results apply to the other cases.

To calculate the visibility, we need the probability density of the phase noise [see (3*b*)], but  determines  only up to a random phase α. Therefore, given (29), the Fourier transform of the phase noise is

and

where  is the amplitude, and we are still free to choose the probability density of α.

By taking α uniform in the  interval,  is the arcsin noise whose effect on the interference visibility is shown in Fig. 2 ▸. Then, the probability density of the phase noise (see the supporting information) is

where ,  and

is the variance.

Finally, by application of (3*b*), the dependence of the visibility Γ on the frequency Ω of the seismic noise is

where  is the Bessel function of the first kind and zero order.

To help the understanding and experimental verifications, it is convenient to examine the visibility as a function of the interferometer displacement  [see (5*a*) and (9*a*)]. Hence, in (34),  and

where we introduce the dimensionless standard deviation and frequency,  and , respectively, and the diffracting-plane spacing *d*.

The result is shown in Fig. 9 ▸. The vertical sections, where  constant, reproduce the green line (dot–dashed) shown in Fig. 2 ▸, where the abscissa is scaled by the interferometer response to the (sinusoidal) displacement. The horizontal sections, where  constant, reproduce the phase frequency response to the interferometer displacement , which is shown in Fig. 4 ▸ (top) as a function of the acceleration . They are modulated by the Bessel function , which maps the rising and falling of the phase response to the interferometer displacement into the visibility, as shown in Fig. 2 ▸.

The visibility dependence on ω is periodic, with periodicity . The null response at  (see Fig. 4 ▸) translates into peaks of maximum visibility. In this case, the oscillations of the interferometer crystals perceived by the neutrons are in phase, so no differential displacement is seen.

The visibility map is symmetric about . In this case, the splitter and mirrors are perceived to oscillate in counter-phase, and the splitter and analyser in phase. The differential displacement of the interferometer crystals is maximum but its amplitude depends on the frequency, as shown in Fig. 4 ▸.

Unfortunately, from the viewpoint of an experimental test, the cutoff frequency  is in the many kilohertz region for any practical interferometer geometry, as shown in Table 1 ▸.

## Appendix B List of the main symbols

Symbols have been defined at their first occurrence. However, to prevent readers from having to go back and forth to search for the definitions, this appendix provides the essential ones.

– reciprocal vector (modulus).

*d* – diffracting-plane spacing.

– splitter, mirrors’ and analyser thickness.

– time of flight through the splitter, mirrors and analyser.

– mirrors’ distances from the splitter and analyser.

– transit times from splitter to mirrors and analyser.

– motion of the *X* crystal.

– diffracting-plane displacement (perceived deformation of the *X* crystal).

– interferometer displacement (monolithic).

– displacement of the first interferometer block (split crystals).

– displacement of the second interferometer block (split crystals).
